# The P450 gene CYP749A16 is required for tolerance to the sulfonylurea herbicide trifloxysulfuron sodium in cotton (*Gossypium hirsutum* L.)

**DOI:** 10.1186/s12870-018-1414-2

**Published:** 2018-09-10

**Authors:** Gregory N. Thyssen, Marina Naoumkina, Jack C. McCarty, Johnie N. Jenkins, Christopher Florane, Ping Li, David D. Fang

**Affiliations:** 1 Cotton Fiber Bioscience Research Unit, USDA-ARS-SRRC, New Orleans, LA 70124 USA; 2 Cotton Chemistry and Utilization Research Unit, USDA-ARS-SRRC, New Orleans, LA 70124 USA; 3 Genetics & Sustainable Agriculture Research Unit, USDA-ARS, Mississippi State, MS 39762 USA

**Keywords:** Acetohydroxyacid synthase, Acetolactate synthase, Cotton, Herbicide tolerance, Trifloxysulfuron sodium (TFS)

## Abstract

**Background:**

Weed management is critical to global crop production and is complicated by rapidly evolving herbicide resistance in weeds. New sources of herbicide resistance are needed for crop plants so that applied herbicides can be rotated or combined to thwart the evolution of resistant weeds. The diverse family of cytochrome P450 proteins has been suggested to be a source of detoxifying herbicide metabolism in both weed and crop plants, and greater understanding of these genes will offer avenues for crop improvement and novel weed management practices.

**Results:**

Here, we report the identification of CYP749A16 (Gh_D10G1401) which is responsible for the natural tolerance exhibited by most cotton, *Gossypium hirsutum* L., cultivars to the herbicide trifloxysulfuron sodium (TFS, CGA 362622, commercial formulation Envoke). A 1-bp frameshift insertion in the third exon of CYP749A16 results in the loss of tolerance to TFS. The DNA marker designed from this insertion perfectly co-segregated with the phenotype in 2145 F_2_ progeny of a cross between the sensitive cultivar Paymaster HS26 and tolerant cultivar Stoneville 474, and in 550 recombinant inbred lines of a multi-parent advanced generation inter-cross population. Marker analysis of 382 additional cotton cultivars identified twelve cultivars containing the 1-bp frameshift insertion. The marker genotypes matched perfectly with phenotypes in 188 plants from the selected twelve cultivars. Virus-induced gene silencing of CYP749A16 generated sensitivity in the tolerant cotton cultivar Stoneville 474.

**Conclusions:**

CYP749A16 located on chromosome D10 is required for TFS herbicide tolerance in cotton. This finding should add to the repertoire of tools available to farmers and breeders for the advancement of agricultural productivity.

**Electronic supplementary material:**

The online version of this article (10.1186/s12870-018-1414-2) contains supplementary material, which is available to authorized users.

## Background

The development of no-till farming has greatly increased overall efficiency of crop production by eliminating the need to expend resources on physically overturning the soil [1]. However, tillage is historically important to weed management, and competition from weeds can reduce crop yields significantly [1]. Herbicides that crops can tolerate, but weeds cannot, are tools for no-till weed management. In some cases, a transgene is introduced into the crop to provide resistance to an otherwise widely effective herbicide [2, 3]. However, eventually weeds gain herbicide resistance, either by gene flow from sexually compatible crop species, or by natural mutation and selection [4, 5]. The identification of novel genetic mechanisms to achieve herbicide resistance or tolerance in crops is one way of circumventing the continually emerging sources of resistance in weeds, and maintaining and advancing overall crop production efficiency.

Cotton, *Gossypium hirsutum* L., is globally the most important source of fibers for textiles [6]. Control of broadleaf weeds such as morningglory (*Ipomoeapurpurea* L. Roth), sicklepod (*Cassia obtusifolia* L.), and pigweed (*Amaranthus retroflexus* L.) with inhibitors of acetolactate synthase (ALS), is based on the tolerance of cotton plants to such herbicides [7]. Sulfonylurea herbicides, including trifloxysulfuron sodium (TFS, CGA 362622, commercial name Envoke), non-competitively bind to ALS and inhibit the synthesis of the branched-chain amino acids [8, 9]. Resistance to such herbicides in several plant species has been attributed to certain non-synonymous changes to amino acid sequences near the herbicide binding site following mutagenesis or selection, including in *Zea mays* [10], *Arabidopsis thaliana* [11], *Beta vulgaris* [12], *Brassica napus* [13], *Glycine max* [14], *Nicotiana tabacum* [15], *Triticum aestivum* [16], *Lolium rigidum* [17], *Alopecurus myosuroides* [18], and *Gossypium hirsutum* [19]. Such mutations allow the ALS protein to function in the presence of the herbicide [8]. The man-made mutations identified in allotetraploid cotton were located in either the A or D subgenome copies of the ALS gene [19, 20]. In cotton, a high level of herbicide resistance was achieved by stable over-expression of an altered ALS sequence [21].

Most cotton cultivars can recover after application of ALS-inhibiting herbicides, even though the ALS allele is sensitive. Tolerance in commercial cultivars does not depend on a transgene or point mutations in the ALS gene [22, 23]. Instead, tolerance of cotton to sulfonylurea herbicides is thought to be due to reduced absorption and translocation of the herbicide in conjunction with rapid metabolism [7]. Cotton leaves absorb less ^14^C-radiolabled TFS than sensitive plants like peanut (*Arachis hypogaea*), and the herbicide was more rapidly metabolized [7, 24].

Non-target site resistance (NTSR) is an important mechanism of herbicide resistance in weeds that often involves P450 proteins [25]. One report identified the mechanism of resistance to phenylurea herbicides as a cytochrome P450 monooxygenase protein [26]. This P450 protein was identified in Jerusalem artichoke (*Helianthus tuberosus*) and conferred resistance to several phenlyurea herbicides when over-expressed in *Arabidopsis thaliana* [27]. The resistance mechanism was shown to be metabolism of the herbicides by the P450 protein [27] which had already been shown in *Zea mays* [28, 29] and *Triticum aestivum* [30]. Nicosulfuron and bentazon-sensitive *Z. mays* lines GA209 and W703a were found to contain the same 392-base pair insertion in a P450 gene, NSF1/BEN1 [31–33]. Another cytochrome P450 gene, CYP81A6, was identified in *Oryza sativa* and shown to provide resistance to sulfonylurea herbicides [34]. Several studies have attributed resistance to the P450 family of proteins based on the observation that co-application of the pesticide malathion, a P450 inhibitor, results in loss of resistance or reduction in tolerance [17, 25, 35, 36]. Due to limited genomic resources in weed species, cloning of these putative NTSR genes was not practical [35]. The P450 protein family comprises hundreds of genes in *A. thaliana* [37]. The draft genome of *G. hirsutum* contains 622 annotated cytochrome P450 genes [38].

Our earlier discovery of an TFS-sensitive cotton cultivar, Paymaster HS26, made possible this study of the genetic basis of sulfonylurea herbicide tolerance in *G. hirsutum* [39]. We previously determined that Paymaster HS26 has identical ALS sequences to tolerant cultivars, and found that a single locus on Chr D10 (also called Chromosome 20) was responsible for tolerance in a segregating F_2_ population [39]. Here, we sequenced sensitive and tolerant recombinant inbred lines (RILs) of a multiparent advanced generation intercross (MAGIC) population, increased the size of the F_2_ population, and screened a diverse panel of cultivated cotton varieties to link a 1-bp frameshift insertion in a cytochrome P450 gene, CYP749A16 (Gh_D10G1401) to theTFS herbicide sensitivity. We further confirmed the role of CYP749A16 in cotton by using virus induced gene silencing (VIGS) to generate sensitivity in a tolerant cotton line.

## Results

### Segregation of TFS herbicide tolerance in the F_2_ and MAGIC populations

Two cotton (*G. hirsutum*) cultivars, Stoneville 474 (STV474) and Paymaster HS26 (HS26) were selected based on our observation of TFS sensitivity in HS26 [39]. STV474, like most cotton cultivars [22], is only transiently damaged by TFS herbicide application, and fully recovers in less than a week in field conditions [39]. To identify the genomic location of the gene responsible for TFS sensitivity in HS26, we developed a new F_2_ population by crossing HS26 with the tolerant cultivar STV474. In 1695 F_2_ progeny plants, 1255 were tolerant while 440 were sensitive (*X*^2^ = 0.83 ns). This supports our previous conclusion that the TFS herbicide tolerance in cotton is conferred by a single dominant locus [39]. Among the eleven parents of the MAGIC population, only HS26 was sensitive. Of the 550 MAGIC RILs, twenty were sensitive.

### Identification of a mutant allele of CYP749A16

**Fig. 1:**
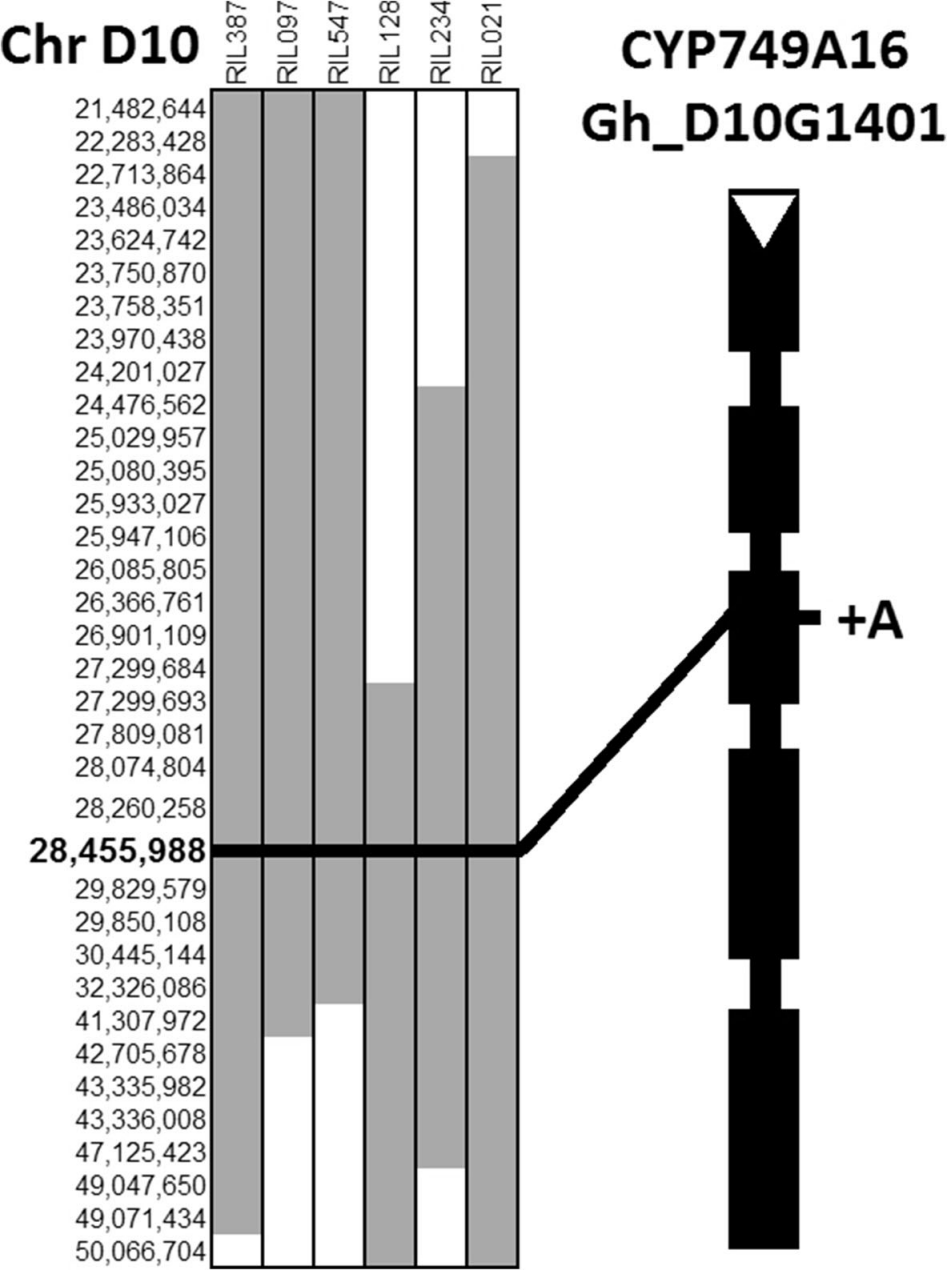
Identification of a frameshift mutation in CYP749A16 (Gh_D10G1401). Polymorphisms that are specific to the trifloxysulfuron sodium (TFS) herbicide sensitive parent, *Gossypium hirsutum* cv Paymaster HS26 were detected in recombinant inbred lines from a MAGIC population by whole genome skim sequencing and are shown in grey, while the reference allele is white. The exon and intron structure of the candidate gene is shown with thick and thin black lines, respectively, and the location of the extra adenine in sensitive plants is labeled

A MAGIC population was developed through a half-diallel crossing scheme between eleven *G. hirsutum* cultivars that included both HS26 and STV474 [40]. The MAGIC population of 550 RILs and their eleven parents were sequenced with short reads. We found polymorphisms that were specific to HS26 and the twenty sensitive RILs in the vicinity of our previously reported locus and searched for their presence in all 550 RILs. One of these, at position 28,455,988 on chromosome D10, is an insertion of an additional adenine in the third exon of a P450 gene, CYP749A16 (Gh_D10G1401) (Fig. 1). We designed a PCR-based marker (Additional file 1: Table S1) and tested for the presence of the frameshift insertion in all 550 RILs, along with 2145 F_2_ plants derived from a cross between HS26 and STV474, and also 382 additional cotton cultivars. In the segregating F_2_ and MAGIC populations, only plants that were homozygous for the disrupted allele were sensitive to TFS herbicide. Of the 382 cultivars tested, the presence of the mutant allele was identified in 12 cultivars (Additional file 1: Table S2). Nine to 23 plants from each of these 12 cultivars were grown in a greenhouse, genotyped at the Gh_D10G1401 locus, and sprayed with TFS herbicide. All plants from cultivars that were homogeneous at the locus were sensitive to TFS herbicide. Seeds from cultivars that were heterogeneous at the locus, based on the initial screen of pooled DNA from multiple individuals of the same cultivar, produced sensitive and tolerant plants. The sensitivity of individually phenotyped plants to the herbicide was in perfect accord with their genotypes at the Gh_D10G1401 locus. Pedigree analysis suggested that *G. hirsutum* cultivar Kekchi is a common ancestor to the sensitive lines [41].

### Response of CYP749A16 to TFS herbicide

**Fig. 2:**
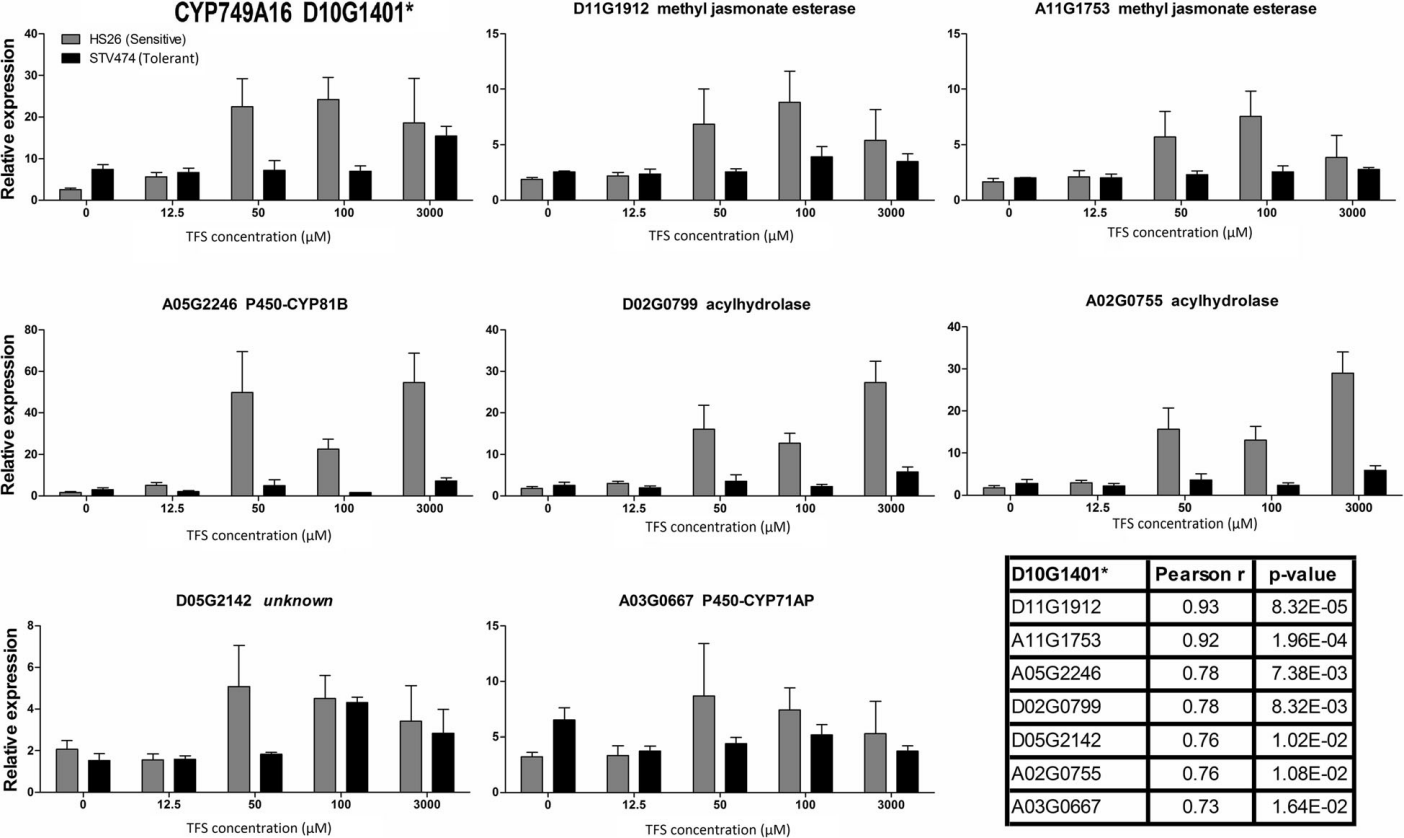
Response of select genes to TFS application in tolerant and sensitive cotton cultivars. Two-week old cotton plants were exposed to 0, 12.5, 50, 100 or 3000 μM TFS. The tolerant cultivar is STV474 and is shown with black bars, while the sensitive HS26 is grey. Error bars represent standard deviation from six biological replicates. The table shows Pearson’s pairwise correlation in expression patterns between tested genes and the TFS tolerance gene, CYP749A16 (Gh_D10G1401). See also Additional file 3: Figure S2

**Fig. 3:**
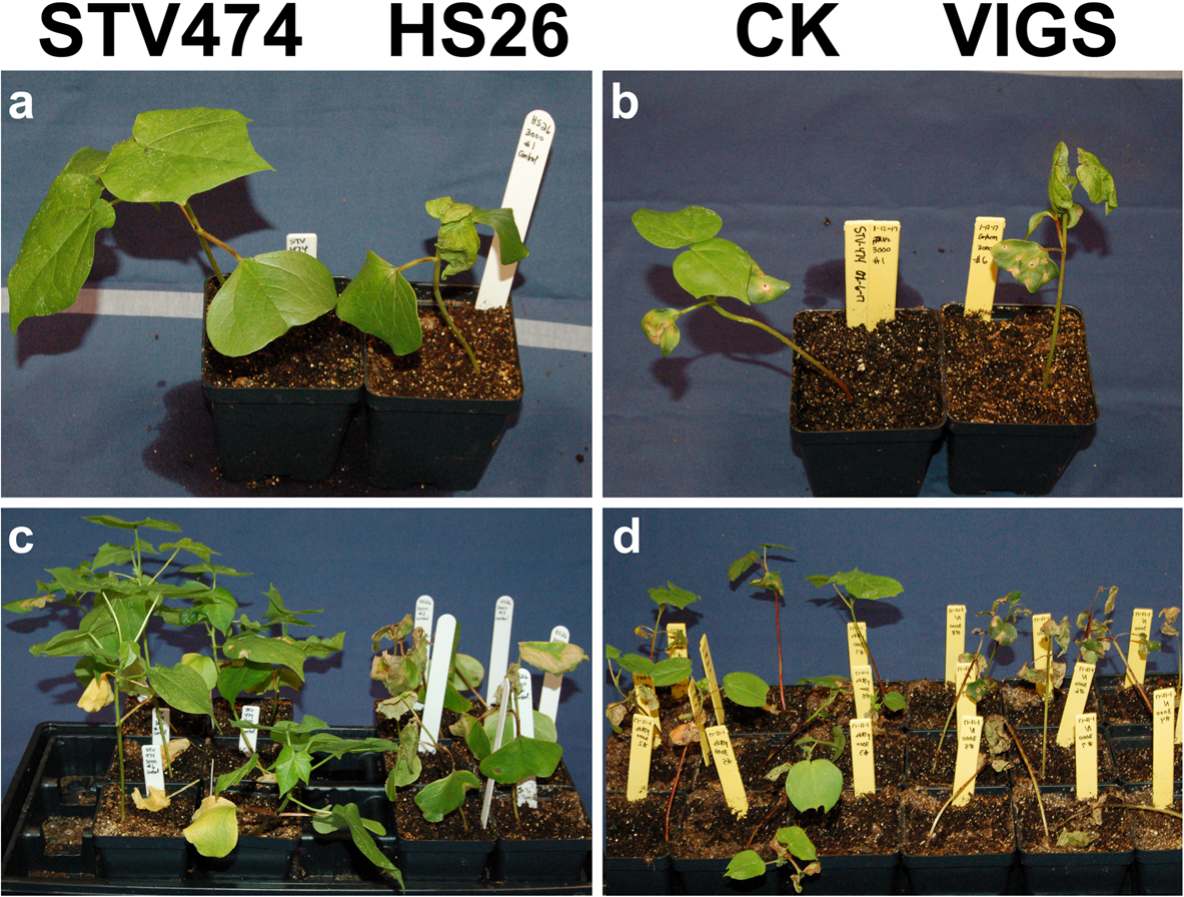
Response of tolerant, sensitive and VIGS cotton plants to TFS herbicide. Three week old cotton plants shown one week after application of 3000 μM TFS herbicide. **a**, **c** Tolerant (STV474) and sensitive (HS26) cultivars. **b**, **d** Tolerant STV474 plants were infiltrated with the helper vector, pTRV1, and either an empty pTRV2 vector (CK) or with a pTRV2-D10G1401 construct to test the effect of virus-induced gene silencing (VIGS) of the CYP749A16 (Gh_D10G1401) gene on TFS tolerance. Damage to cotyledons was due to the VIGS infiltration, while damage to true leaves could be attributed to the induced TFS sensitivity

We compared the dose dependent induction of CYP749A16 transcripts in STV474 and HS26 (Fig. 2). At concentrations of 12.5 μM TFS and below, the gene was only weakly expressed in each line. At and above 50 μM TFS, CYP749A16 transcripts were induced in the sensitive line HS26, but CYP749A16 transcripts were only significantly induced at 3000 μM TFS in the tolerant line, STV474. At this concentration the STV474 plants quickly recovered from any damage, while the HS26 plants withered and died (Fig. 3a, c).

### Virus-induced gene silencing (VIGS) of CYP749A16

To confirm the involvement of CYP749A16 in tolerance to TFS herbicide with an independent line of evidence, we constructed a virus induced gene silencing vector based on the tobacco rattle virus to suppress gene expression in tolerant STV474 plants. We optimized the specificity of the targeting region (See Methods and Additional file 2: Figure S1, Additional file 1: Table S3) and cloned 350-bp of coding sequence for the reference Gh_D10G1401 gene into the pTRV2 vector (Additional file 1: Table S4). A week after infiltration, when new tissues had emerged including two true leaves, we applied TFS herbicide at 3000 μM, the rate that we found to induce CYP749A16 expression in STV474 plants (Fig. 2). One week later, the true leaves of the VIGS plants withered and died, while the control plants had no obvious damage to their true leaves (Fig. 3b, d). The cotyledons of both the control and VIGS plants were damaged during the infiltration procedure.

### Co-expression analysis

To explore the possibility that more genes from cotton, beyond CYP749A16, are involved in generating TFS herbicide tolerance, we examined expression of select genes by RT-qPCR. We queried the ccNET database with Gh_D10G1401 to find genes that are often co-expressed [42]. From the list, we selected genes with the highest mutual ranks, plus high ranking P450 genes and genes with no known ortholog in *Arabidopsis*. We tested twenty genes in STV474 and HS26 plants that had been challenged with different concentrations of TFS herbicide. Of these, seven showed significant positive correlation (> 0.7) (Fig. 2) while thirteen showed lower or negative correlation (< 0.7) with Gh_D10G1401 (Additional file 3: Figure S2).

## Discussion and conclusions

We previously identified sensitivity to the TFS herbicide in cotton cultivar Paymaster HS26 and demonstrated that this was not due to mutations in the acetolactate synthase (ALS) gene that is the target of the herbicide [39]. Here, we have shown that the gene responsible for TFS tolerance in cotton is a cytochrome P450 gene, CYP749A16 (Gh_D10G1401) and that sensitive cotton cultivars carry a 1-bp frameshift insertion in the third exon of the gene. We are able to conclude that CYP749A16 is necessary for TFS tolerance in cotton, by the co-segregation or association of the frameshift insertion in segregating and cultivated populations (Fig. 1 and Additional file 1: Table S2). This was further confirmed by our observation that VIGS of CYP749A16 was able to induce sensitivity to TFS in a tolerant variety (Fig. 3). Since related P450 genes have been shown to confer herbicide resistance by catalyzing chemical modifications to herbicides, it is not surprising to have found that a P450 gene is necessary for TFS tolerance in cotton. Indeed cotton cultivars, including STV474, were reported to exhibit increased damage when TFS is mixed with malathion, a P450 inhibitor, which could be taken as evidence of P450 mediated metabolism of the TFS herbicide in cotton [43, 44].

The cytochrome P450 family comprises 622 genes in *G. hirsutum*, and some plant species have experienced lineage-specific expansions and losses of subfamilies [45, 46]. Of particular interest is the loss of the CYP749 family from the Brassicales which includes *Arabidopsis*, despite their presence in the sister group Malvales, which includes cotton, and most other rosids [46]. While we have demonstrated that CYP749A16 is required for TFS tolerance in cotton, further work is required to determine if it is sufficient to generate resistance in another plant species. We began the search for additional proteins that may also be involved in TFS detoxification by examining the expression of select genes that are often co-expressed with CYP749A16. We found that some of the select genes were similarly induced by TFS as CYP749A16 (Fig. 2), but others were not (Additional file 3: Figure S2). Further, more exhaustive expression analysis should be conducted in the future.

There does not appear to be a functional A-subgenome homeolog of Gh_D10G1401, in the reference genome we used or cultivars we studied [38]. At the homeologous location on Chr A10 is a sequence with high homology to Gh_D10G1401, but it is missing an adenine residue at A10:54,681,293, which results in a frameshift mutation, rendering this sequence an unannotated pseudogene (Additional file 1: Table S6). This observation helps explain the simple recessive nature of the TFS sensitivity locus in an allotetraploid crop.

Our earlier genetic mapping of the TFS-tolerance locus placed CYP749A16 outside of the candidate interval by 3.5 Mb [39]. That work relied on twelve SSR-markers and 450 F_2_ progeny, while here we used whole genome sequencing of 550 RILs from an eleven-parent MAGIC population to identify the frameshift mutation, which we then found to be completely linked in 2145 F_2_ progeny and predictive in a diversity panel of 384 varieties. This demonstrates the importance, in any genetic mapping project, to eventually find the causative mutation, rather than simply flanking markers. The marker for the causative mutation we describe here could be used by cotton breeders to eliminate TFS sensitivity from cotton cultivars, or to deliberately select for sensitivity, which may be useful in some crop-rotation systems. It is possible that transgenic introduction of CYP749A16 to other crops would confer TFS resistance which would add to the tools available to breeders and farmers for no-till weed management.

## Methods

### Plant materials

Crosses between *G. hirsutum* cv. Paymaster HS26 (HS26) and *G. hirsutum* cv Stoneville 474 (STV474) were made to establish the F_1_ population, which was self-pollinated to produce the F_2_. The F_2_ seeds were planted in the Plant Science Research Farm at Mississippi State, MS, USA. During the plant growing season, standard conventional field practices were followed. In addition to the 450 F_2_ individuals previously studied [39], we genotyped and phenotyped an additional 1695 F_2_ individuals in 2014 in the same way, for the present study.

A MAGIC population was developed through a half-diallel crossing scheme between eleven parents that included both HS26 and STV474 [40]. The 55 F_1_ were considered as 55 half-sib families and designated as Cycle 0 (C_0_). Five cycles (C_1_ to C_5_) of random mating were made by bulking an equal amount of pollen from each of the 55 families. After that, the plants were self-pollinated for six generations using single seed descent to produce 550 RILs [40, 47]. The eleven parents and 550 RILs were planted in the Plant Science Research Farm at Mississippi State, MS, USA in 2010. Each RIL was planted in two 12 m plots consisting of about 120 plants per replicate. Of the 550 RILs, twenty were sensitive to TFS.

Three hundred eighty-four cotton cultivars, including HS26 and STV474, (Additional file 1: Table S2) that had been included in the National Cotton Variety Trials (NCVT) over the past sixty years were used to create a diversity panel. For each cultivar, young leaves from about ten seedlings were collected for DNA extraction.

### Field TFS herbicide treatment

The parental cultivars, F_2_ plants and MAGIC RILs were subjected to over-the-top application of TFS along with the 25% *w*/w additional proprietary inactive ingredients in the commercially available herbicide formulation, Envoke (Syngenta Crop Protection Inc., Greensboro, NC, USA) six weeks after planting at a rate of 10.5 g TFS/ha and volume of 186.8 l/ha (equivalent to 100 μM TFS). The phenotypes were observed as tolerant or sensitive based on observations of damage to plants one and two weeks later.

### Greenhouse TFS herbicide treatment

Out of the 384 cultivars comprising the diversity panel, thirteen including Paymaster HS26 had a sensitive or heterogeneous genotype at the Gh_D10G1401 locus (Additional file 1: Table S2). Except HS26, the other twelve cultivars were planted in 5-gal pots in a greenhouse in New Orleans, LA, USA in January, 2016. Nine to 23 seedlings were tested for each cultivar. Six-week-old seedlings were sprayed over-the-top with Envoke herbicide (100 μM TFS). Before herbicide treatment, a young leaf was collected from each individual plant for DNA extraction. A total of 188 seedlings were used.

### Growth chamber TFS herbicide treatment

STV474 and HS26 seeds were first germinated in a greenhouse. After emergence, seedlings were moved into a 22 °C growth chamber under a 12-h light and 12-h dark cycle. After two weeks, at the complete expansion of two true leaves, plants were sprayed with different concentrations of Envoke herbicide (0, 12.5, 50, 100 or 3000 μM TFS). Each treatment group contained six plants. Leaf tissues were collected one week later for RNA isolation that would be used for RT-qPCR.

### DNA and RNA isolation

Young leaves were collected from parental cultivars, 2145 F_2_ progeny, 550 MAGIC RILs, 384 NCVT cultivars, and 188 greenhouse-grown plants from a subset of 12 cultivars. DNA was isolated as described previously [48]. RNA was isolated from leaves according to the procedures used before [49].

### Genome sequence analysis

The whole genomes of eleven MAGIC parents were sequenced at 20× coverage and all 550 RILs were sequenced at 3× coverage with Illumina short read (101 or 150-bp) paired-end sequencing with 300 to 500-bp inserts. Genome sequencing was conducted by Novogene Corporation (Chula Vista, CA, USA). Sequence reads were aligned to the NBI *G. hirsutum* cv TM-1 reference genome [38] with GSNAP software [50]. Variants were identified with samtools and bcftools software [51]. We filtered the variants to find polymorphisms that were unique to HS26 and shared by all twenty TFS herbicide sensitive RILs. Among such polymorphisms was an additional adenosine residue after position 28,455,988 on Chr D10 (Fig. 1). This was the only such polymorphism detected in the coding sequence of a gene. Since this mutation would result in a frameshift disruption of the P450 gene CYP749A16 (Gh_D10G1401), we designed a PCR-based marker to detect this allele. The primers (Additional file 1: Table S1) were used to detect the insertion allele in all the plant materials used in this study. The marker was run in a SYBR master mix on a BioRad CFX qPCR machine (Hercules, CA, USA) so that observations of Ct values could be used to determine presence or absence of amplicons, as before [52].

### Virus-induced gene silencing

A 350-bp fragment of CYP749A16 was synthesized based on the reference sequence (Additional file 1: Table S4) [38]. The site was chosen by SGN VIGS Tool software [53] using the NBI reference genome [38] (Additional file 2: Figure S1 and Additional file 1: Table S3). This fragment was cloned into *Eco*RI–*Kpn*I digested pTRV2 to produce a VIGS vector named pTRV2-D10G1401. The pTRV1 helper plasmid, pTRV2- D10G1401, along with VIGS positive control pTRV2–Cla1 and negative control empty vector pTRV2–0 were introduced into the *Agrobacterium* strain GV3101 by electroporation (Bio-Rad, Hercules, CA, USA). A previously published protocol was used for the VIGS assay [54]. Briefly, the transformed *Agrobacterium* colonies were grown overnight at 28 °C in an antibiotic selection medium containing kanamycin 50 mg/L and gentamycin 25 mg/L. *Agrobacterium* cells were collected and re-suspended in infiltration medium (10 mM MgCl_2_, 10 mM MES and 200 μM acetosyringone), adjusted to OD600 = 1.5. *Agrobacterium* strains containing pTRV1 and pTRV2 vectors were mixed at a ratio of 1:1. Seedlings of STV474 with mature cotyledons but without a visible true leaf (seven days after germination) were infiltrated by inserting the *Agrobacterium* suspension into the cotyledons via a syringe. Each treatment group contained nine plants. The plants were grown in pots at 22 °C in a growth chamber under a 12-h light and 12-h dark cycle. After two weeks, plants were sprayed with herbicide at the concentration of 3000 μM TFS (Fig. 3b, d).

### Co-expression analysis

The ccNet database was queried for co-expressed genes of Gh_D10G1401 [42]. Twenty genes, including four P450 genes, and eight genes without an obvious ortholog in *Arabidopsis* were selected for analysis and specific RT-qPCR primers were designed (Additional file 1: Table S5). Expression was tested on the STV474 and HS26 plants that had been exposed to a range of TFS concentrations. Pearson correlation coefficients and *p*-values were calculated to identify genes that were co-expressed with Gh_D10G1401 in response to TFS application (Fig. 2 and Additional file 3: Figure S2).

## Additional files

Additional file 1: **Table S1.** qPCR Genetic marker primers used in this study. **Table S2.** List of 384 cotton cultivars included in the diversity panel and their genotype at the Gh_D10G1401 locus. **Table S3.** Specificity of gene fragment for VIGS in NBI reference sequences. **Table S4.** Gene fragment for VIGS. **Table S5.** RT-qPCR Primers used in this study. **Table S6.** Multiple alignment of Gh_D10G1401 from Paymaster HS26 and wild-type cotton, including potential homeologous region on Chr A10. (DOCX 38 kb)

Additional file 2: **Figure S1.** Design of VIGS target sequence. The SGN VIGS Tool software selected the 350-bp targeting sequence (yellow region) from the NBI reference genome for cotton, *Gossypium hirsutum* cv. TM-1. The full length coding sequence of Gh_D10G1401 was chopped into all 1542 possible 21-mers. These 21-mers were aligned to annotated coding sequences, allowing one mismatch. Off target alignments of 21-mers are shown in red. (JPG 683 kb)

Additional file 3: **Figure S2.** Response of additional genes to TFS application in tolerant and sensitive cotton cultivars. Two-week old cotton plants were exposed to 0, 12.5, 50, 100 or 3000 μM TFS. The tolerant cultivar is STV474 and is shown with black bars, while the sensitive HS26 is grey. Error bars represent standard deviation from six biological replicates. The table shows Pearson’s pairwise correlation in expression patterns between tested genes and CYP749A16 (Gh_D10G1401). See also Fig. 2. (JPG 5645 kb)
