# Supplementary material for: The P450 gene CYP749A16 is required for tolerance to the sulfonylurea herbicide trifloxysulfuron sodium in cotton (Gossypium hirsutum L.)
Source: BMC Plant Biol. 2018 Sep 10;18:186. doi: 10.1186/s12870-018-1414-2 (PMC6131939; doi:10.1186/s12870-018-1414-2)
Supplement: Supplementary file 1 — Table S1. qPCR Genetic marker primers used in this study. Table S2. List of 384 cotton cultivars included in the diversity panel and their genotype at the Gh_D10G1401 locus. Table S3. Specificity of gene fragment for VIGS in NBI reference sequences. Table S4. Gene fragment for VIGS. Table S5. RT-qPCR Primers used in this study. Table S6. Multiple alignment of Gh_D10G1401 from Paymaster HS26 and wild-type cotton, including potential homeologous region on Chr A10. (DOCX 38 kb) [file 12870_2018_1414_MOESM1_ESM.docx]

**Table S1. qPCR Genetic marker primers used in this study.** These primers were used to determine the genotype of a 1-bp insertion in Gh_D10G1401.

| D10_2845_wtF | TTCTTGAATTCACAAGGACTTATAG |
| --- | --- |
| D10_2845_muF | TTCTTGAATTCACAAGGACTTATAA |
| D10_2845_R2 | TCATTTTGTTAATTTGTTTGTTGC |
| D10_2845_R1 | TTATTTCTGGAAACTAGAAGTTACAC |

**Table S2. List of 384 cotton cultivars included in the diversity panel and their genotype at the Gh_D10G1401 locus.** For each cultivar, DNA was pooled from ten individuals. Homogenous detection of the reference allele is “A” and of the sensitive allele first detected in Paymaster HS26 is “B”. Detection of both alleles is “H”. When plants from the “H” cultivars were examined individually, most plants were “A” or “B” indicating heterogeneity in the cultivars. When tested individually, all plants with the “B” genotype were sensitive to TFS herbicide.

| **#** | **Variety Name** | **genotype of Gh_D10G1401** |
| --- | --- | --- |
| 1 | 94 WD-17 | A |
| 2 | 94L 25 | A |
| 3 | ACALA 1517 BR-1 | A |
| 4 | ACALA 1517 BR-2 | A |
| 5 | ACALA 1517 SR-1 | A |
| 6 | ACALA 1517 SR-2 | A |
| 7 | ACALA 1517 SR-3 | A |
| 8 | ACALA 1517-70 | A |
| 9 | ACALA 1517-75 | A |
| 10 | ACALA 1517-77 | A |
| 11 | ACALA 1517-88 | A |
| 12 | ACALA 1517-95 | A |
| 13 | ACALA 1517-99 | A |
| 14 | ACALA 1517-D | A |
| 15 | ACALA 1517-E2 | A |
| 16 | ACALA 1517-V | A |
| 17 | ACALA 1755-91 | A |
| 18 | ACALA 4-42 | A |
| 19 | ACALA 4-42-1958 E | A |
| 20 | ACALA GC 510 | A |
| 21 | ACALA MAXXA | A |
| 22 | ACALA PREMA | A |
| 23 | ACALA ROYALE | A |
| 24 | ACALA SJ-1 | A |
| 25 | ACALA SJ-2 | A |
| 26 | ACALA SJ-3 | A |
| 27 | ACALA SJ-4 | A |
| 28 | ACALA SJ-5 | A |
| 29 | ACALA SJC-1 | A |
| 30 | ACALA ULTIMA | A |
| 31 | AK 1264 | A |
| 32 | ALL TEX ATLAS | H |
| 33 | AR 9101-91-09 | A |
| 34 | AR 9304-39-15 | A |
| 35 | AR 9308-27-04 | A |
| 36 | AR 9314-24-16 | A |
| 37 | AR 9704-13-05 | A |
| 38 | AR 9704-13-08 | A |
| 39 | ARK 0009-13-01 | A |
| 40 | ARK 0015-06-09 | A |
| 41 | ARK 0102-48 | A |
| 42 | ARK 8712 | A |
| 43 | ARK 9610 | A |
| 44 | ARK 9803-23-04 | A |
| 45 | ARK UGO | A |
| 46 | ATLAS (AXC) 261 | A |
| 47 | ATLAS (AXDK) | A |
| 48 | ATLAS (CXE) 352 | A |
| 49 | ATLAS 66 | A |
| 50 | ATLAS 67 | A |
| 51 | AUBURN 56 | A |
| 52 | AUBURN M | A |
| 53 | AUSTIN | A |
| 54 | AxTE | A |
| 55 | AZ 64 | A |
| 56 | B49-20 | A |
| 57 | B7465 | A |
| 58 | BAYOU 7769 | A |
| 59 | BLIGHTMASTER | A |
| 60 | BLIGHTMASTER A-5 | A |
| 61 | BOBSHAW 54 | A |
| 62 | BR 110 | A |
| 63 | BS&D UTE | A |
| 64 | CAROLINA QUEEN | A |
| 65 | CASCOT C-13 | A |
| 66 | CASCOT L-7 | A |
| 67 | CENCOT | A |
| 68 | COKER 100A | A |
| 69 | COKER 118-6903 | A |
| 70 | COKER 124 | A |
| 71 | COKER 130 | A |
| 72 | COKER 139 | A |
| 73 | COKER 201 | A |
| 74 | COKER 208 | A |
| 75 | COKER 304 | A |
| 76 | COKER 310 | A |
| 77 | COKER 312 | A |
| 78 | COKER 3131 | A |
| 79 | COKER 315 | A |
| 80 | COKER 320 | A |
| 81 | COKER 4104 | A |
| 82 | COKER 413 | A |
| 83 | COKER 413 8913 | A |
| 84 | COKER 417 | A |
| 85 | COKER 420 | A |
| 86 | COKER 423 | A |
| 87 | COKER 423-70911 | A |
| 88 | COKER 504 | A |
| 89 | COKER 5110 | A |
| 90 | COKER 71105 | A |
| 91 | COKER 71500 | A |
| 92 | COKER 71-511 | A |
| 93 | COKER 801N | A |
| 94 | COKER 8103 | A |
| 95 | COKER 81-102 | A |
| 96 | COKER 81-613 | A |
| 97 | COKER 8215 | A |
| 98 | COKER 82-211 | A |
| 99 | CP 0803 | A |
| 100 | CP 820589 | A |
| 101 | D & PL FOX 4 | A |
| 102 | DEKALB 108 | A |
| 103 | DEL CERRO | A |
| 104 | DELCOT 277 | A |
| 105 | DELCOT 311 | A |
| 106 | DELCOT 344 | A |
| 107 | DELFOS 9169 | A |
| 108 | DELTA PEARL | A |
| 109 | DELTA QUEEN | A |
| 110 | DELTAPINE 1032DF | A |
| 111 | DELTAPINE 1048DF | A |
| 112 | DELTAPINE 1050DF | A |
| 113 | DELTAPINE 1410DF | A |
| 114 | DELTAPINE 15 | A |
| 115 | DELTAPINE 15A | A |
| 116 | DELTAPINE 16 | A |
| 117 | DELTAPINE 20 | A |
| 118 | DELTAPINE 25 | A |
| 119 | DELTAPINE 26 | A |
| 120 | DELTAPINE 32B | A |
| 121 | DELTAPINE 393 | A |
| 122 | DELTAPINE 41 | A |
| 123 | DELTAPINE 45 | A |
| 124 | DELTAPINE 451 | A |
| 125 | DELTAPINE 458BR | A |
| 126 | DELTAPINE 45A | A |
| 127 | DELTAPINE 491 | A |
| 128 | DELTAPINE 493 | A |
| 129 | DELTAPINE 50 | A |
| 130 | DELTAPINE 51 | A |
| 131 | DELTAPINE 5111 | A |
| 132 | DELTAPINE 523 | A |
| 133 | DELTAPINE 5409 | A |
| 134 | DELTAPINE 5415 | A |
| 135 | DELTAPINE 5415R | A |
| 136 | DELTAPINE 5461 | A |
| 137 | DELTAPINE 55 | A |
| 138 | DELTAPINE 555BR | A |
| 139 | DELTAPINE 5690 | A |
| 140 | DELTAPINE 5826 | A |
| 141 | DELTAPINE 607 | A |
| 142 | DELTAPINE 61 | A |
| 143 | DELTAPINE 62 | A |
| 144 | DELTAPINE 6532 | A |
| 145 | DELTAPINE 655BR | A |
| 146 | DELTAPINE 6582 | A |
| 147 | DELTAPINE 675 | A |
| 148 | DELTAPINE 69 | A |
| 149 | DELTAPINE 70 | A |
| 150 | DELTAPINE 7559-6139 | A |
| 151 | DELTAPINE 77 | A |
| 152 | DELTAPINE 90 | A |
| 153 | DELTAPINE 9056 | A |
| 154 | DELTAPINE 9765 | A |
| 155 | DELTAPINE NSL | A |
| 156 | DELTAPINE SMOOTH LEAF | A |
| 157 | DELTAPINE SR 2 | A |
| 158 | DELTAPINE SR-383 | H |
| 159 | DELTAPINE SR-4 | A |
| 160 | DELTAPINE SR-5 | A |
| 161 | DES 119 | A |
| 162 | DES 1195 | A |
| 163 | DES 24 | A |
| 164 | DES 422 | A |
| 165 | DES 56 | A |
| 166 | DIXIE KING | A |
| 167 | DIXIE KING II | A |
| 168 | DIXIE KING III | A |
| 169 | DUNN 119 | A |
| 170 | DUNN 219 | A |
| 171 | EMPIRE WR | A |
| 172 | EMPIRE WR-61 | A |
| 173 | EXCESS | H |
| 174 | FIBERMAX 1773LL_D | A |
| 175 | FIBERMAX 1845LL_D | A |
| 176 | FIBERMAX 832 | A |
| 177 | FIBERMAX 9058F | H |
| 178 | FIBERMAX 958 | A |
| 179 | FIBERMAX 966 | A |
| 180 | FIBERMAX 989 | A |
| 181 | G&P 1005 | A |
| 182 | G&P 3755 | A |
| 183 | G&P 3774 | A |
| 184 | G&P 5479 | A |
| 185 | GA 90-41 | A |
| 186 | GA 98028 | A |
| 187 | GA 98084 | A |
| 188 | GA HT | A |
| 189 | GC 9033 | A |
| 190 | GEORGIA KING | A |
| 191 | GREGG | A |
| 192 | GREGG 35 | A |
| 193 | GREGG 35W | A |
| 194 | GSA 71 | A |
| 195 | H 1215 | A |
| 196 | H 1220 | A |
| 197 | H 1244 | A |
| 198 | H 1560 | A |
| 199 | HANCOCK | A |
| 200 | HOPICALA | A |
| 201 | HS 44 | A |
| 202 | HS 46 | A |
| 203 | HY 39 | A |
| 204 | IF 1006 | A |
| 205 | JACO 6078 | A |
| 206 | JACO 7164 | A |
| 207 | JACO 7165 | A |
| 208 | JACO 8067 | A |
| 209 | JACO 8073 | A |
| 210 | JAJO 1145 | A |
| 211 | JAJO 3007 | A |
| 212 | JAJO 3077 | A |
| 213 | KC 311 | A |
| 214 | KC 380 | A |
| 215 | KNX99 | A |
| 216 | LA 1511775 | A |
| 217 | LA 830887 | A |
| 218 | LA 870354 | A |
| 219 | LaDASS 5175 | A |
| 220 | LANKART 142 | A |
| 221 | LANKART 57 | A |
| 222 | LANKART 611 | A |
| 223 | LANKART LX 571 | A |
| 224 | LOCKETT 4789 | A |
| 225 | LOCKETT 4789A | A |
| 226 | LOCKETT 77 | A |
| 227 | LOCKETT 88A | A |
| 228 | LOCKETT BXL | A |
| 229 | McNAIR 210 | A |
| 230 | McNAIR 220 | A |
| 231 | McNAIR 235 | A |
| 232 | McNAIR 3034 | A |
| 233 | McNAIR 3150 | A |
| 234 | McNAIR 511 | A |
| 235 | MCNAIR 612 | A |
| 236 | MCNAIR 7215 | A |
| 237 | MCNAIR 8418 | A |
| 238 | McNAIR 9416 | A |
| 239 | MCNAIR 95117 | A |
| 240 | MD 09 NE | A |
| 241 | MD 25 | A |
| 242 | MD 25ne | A |
| 243 | MD 25y | A |
| 244 | MD 51ne | A |
| 245 | MD 82ne | A |
| 246 | MO 61-470 F | A |
| 247 | MO 63-079 | A |
| 248 | MO 63-079 A | A |
| 249 | MO 63-277E | A |
| 250 | MO 63-279D | A |
| 251 | MO 82-137 | A |
| 252 | NC 72 | A |
| 253 | NM 03012 | A |
| 254 | NM 03K1028 | A |
| 255 | NORTHERN STAR 4-11 | A |
| 256 | NuCOTN33B | A |
| 257 | OA 211 | A |
| 258 | PARROTT | A |
| 259 | PAYMASTER 101A | H |
| 260 | PAYMASTER 111 | A |
| 261 | PAYMASTER 111A | A |
| 262 | PAYMASTER 1218BR | A |
| 263 | PAYMASTER 1220R | A |
| 264 | PAYMASTER 145 | A |
| 265 | PAYMASTER 147 | A |
| 266 | PAYMASTER 18 | A |
| 267 | PAYMASTER 202 | H |
| 268 | PAYMASTER 266 | A |
| 269 | PAYMASTER 303 | H |
| 270 | PAYMASTER 330 | H |
| 271 | PAYMASTER 404 | A |
| 272 | PAYMASTER 54B | B |
| 273 | PAYMASTER 909 | A |
| 274 | PAYMASTER 9506-0081 | A |
| 275 | PAYMASTER HS 200 | A |
| 276 | PAYMASTER HS26 | B |
| 277 | PAYMASTER PM 183 | B |
| 278 | PAYMASTER TEJAS | A |
| 279 | PD 0109 | A |
| 280 | PD 0111 | A |
| 281 | PD 0113 | A |
| 282 | PD 1 | A |
| 283 | PD 2 | A |
| 284 | PD 2165 | A |
| 285 | PD 2165B | A |
| 286 | PD 2173 | A |
| 287 | PD 2292 | A |
| 288 | PD 259A | A |
| 289 | PD 3 | A |
| 290 | PD 3246 | A |
| 291 | PD 3-3967 | A |
| 292 | PD 3548 | A |
| 293 | PD 3572 | A |
| 294 | PD 3608 | A |
| 295 | PD 4381 | A |
| 296 | PD 4381-54 | A |
| 297 | PD 4381B | A |
| 298 | PD 4585 | A |
| 299 | PD 5358 | A |
| 300 | PD 5363 | A |
| 301 | PD 5472 | A |
| 302 | PD 5582 | A |
| 303 | PD 6132 | A |
| 304 | PD 6162 | A |
| 305 | PD 6186 | A |
| 306 | PD 695 | A |
| 307 | PD 7396 | A |
| 308 | PD 8619 | A |
| 309 | PD 9222 | A |
| 310 | PD 9223 | A |
| 311 | PD 9232 | A |
| 312 | PD 9363 | A |
| 313 | PD 94035 | A |
| 314 | PD 94045 | A |
| 315 | PD 94063 | A |
| 316 | PHX4912WF | A |
| 317 | PHY 33 | A |
| 318 | PHY367WF | A |
| 319 | PHY375WF | A |
| 320 | PHY499WF | A |
| 321 | PHY565WF | A |
| 322 | PLAINS | A |
| 323 | PSC 355 | A |
| 324 | QUAWPAW | A |
| 325 | QUICKIE | A |
| 326 | RAIDER | A |
| 327 | REX | A |
| 328 | REX SMOOTHLEAF | A |
| 329 | SC-1 | A |
| 330 | SG 105 | A |
| 331 | SG 125 | A |
| 332 | SG 501 | A |
| 333 | SG 747 | A |
| 334 | SOUTHERN STAR 5 | A |
| 335 | SOUTHLAND 400 | A |
| 336 | STONEVILLE 612 3234 | A |
| 337 | STONEVILLE 112 | A |
| 338 | STONEVILLE 151 | A |
| 339 | STONEVILLE 213 | A |
| 340 | STONEVILLE 256 | A |
| 341 | STONEVILLE 302 | H |
| 342 | STONEVILLE 3202 | A |
| 343 | STONEVILLE 42 | A |
| 344 | STONEVILLE 4288DF | A |
| 345 | STONEVILLE 453 | A |
| 346 | STONEVILLE 474 | A |
| 347 | STONEVILLE 4892BR | A |
| 348 | STONEVILLE 506 | A |
| 349 | STONEVILLE 508-9083 | A |
| 350 | STONEVILLE 508-9117 | A |
| 351 | STONEVILLE 580 | A |
| 352 | STONEVILLE 603 | A |
| 353 | STONEVILLE 62 | A |
| 354 | STONEVILLE 7 | A |
| 355 | STONEVILLE 731 N | A |
| 356 | STONEVILLE 7A | A |
| 357 | STONEVILLE 804 | A |
| 358 | STONEVILLE 825 | A |
| 359 | STONEVILLE 907 | A |
| 360 | STONEVILLE LA 887 | A |
| 361 | STRIPPER 31A | A |
| 362 | SV 93 | A |
| 363 | TAM 04WB-33s | A |
| 364 | TAM 06WE-62-4 | A |
| 365 | TAM 96 WD-18 | A |
| 366 | TAMCOT 22 | A |
| 367 | TAMCOT CAMD-E | A |
| 368 | TAMCOT CD3H | A |
| 369 | TAMCOT HQ95 | A |
| 370 | TAMCOT LUXOR | A |
| 371 | TAMCOT PYRAMID | A |
| 372 | TAMCOT SP 21S | A |
| 373 | TAMCOT SP 37 | A |
| 374 | TAMCOT SP 37H | H |
| 375 | TAMCOT SP-21 | A |
| 376 | TAMCOT SPHINX | A |
| 377 | TH 149 | A |
| 378 | TH 149-20 | A |
| 379 | TIDELAND TPSA 69 | A |
| 380 | TIFCOT 56 | A |
| 381 | WESTBURN | A |
| 382 | WESTBURN 70 | A |
| 383 | WESTBURN M | A |
| 384 | WESTERN STORMPROOF | A |

**Table S3. Specificity of gene fragment for VIGS in NBI reference sequences.** A 350-bp targeting sequence of Gh_D10G1401 (Table S4) was analyzed with the SGN VIGS Tool software and the NBI reference genome for cotton, *Gossypium hirsutum* cv TM-1. The targeting sequence was chopped into all 329 possible 21-mers. These 21-mers were aligned to annotated coding sequences in the NBI reference genome, allowing zero, one or two mismatches. The number of 21-mers that match in this way were counted, and is presented in this table. The similar table using the alternative BGI reference genome is Table S4, and graphical presentations of the alignments for 1-mistmach are Figures S1.

| **NBI-Gene** | **0-mismatch** | **1-mismatch** | **2-mismatch** |
| --- | --- | --- | --- |
| Gh_D10G1401 | 329 | 329 | 329 |
| Gh_A02G0223 | 6 | 31 | 54 |
| Gh_D02G0286 | 2 | 4 | 28 |
| Gh_D02G2387 | 0 | 10 | 22 |
| Gh_D02G0285 | 0 | 4 | 18 |
| Gh_A02G0225 | 0 | 3 | 14 |
| Gh_D02G0287 | 0 | 2 | 5 |
| Gh_A02G0222 | 0 | 0 | 20 |
| Gh_A02G0219 | 0 | 0 | 19 |
| Gh_A02G0220 | 0 | 0 | 16 |
| Gh_D13G0628 | 0 | 0 | 13 |
| Gh_A02G0197 | 0 | 0 | 10 |
| Gh_Sca005510G01 | 0 | 0 | 10 |
| Gh_D04G1889 | 0 | 0 | 10 |
| Gh_D02G0289 | 0 | 0 | 8 |
| Gh_D13G0888 | 0 | 0 | 6 |
| Gh_D02G2386 | 0 | 0 | 3 |
| Gh_D13G0688 | 0 | 0 | 3 |
| Gh_A13G0459 | 0 | 0 | 3 |
| Gh_D11G0011 | 0 | 0 | 2 |
| Gh_A11G0014 | 0 | 0 | 2 |
| Gh_A13G1229 | 0 | 0 | 2 |
| Gh_A01G1051 | 0 | 0 | 1 |
| Gh_D04G1314 | 0 | 0 | 1 |
| Gh_D05G3009 | 0 | 0 | 1 |
| Gh_D13G1656 | 0 | 0 | 1 |
| Gh_D02G1039 | 0 | 0 | 1 |
| Gh_A08G1757 | 0 | 0 | 1 |
| Gh_D12G0760 | 0 | 0 | 1 |
| Gh_D12G1392 | 0 | 0 | 1 |
| Gh_D03G1575 | 0 | 0 | 1 |
| Gh_A05G2590 | 0 | 0 | 1 |
| Gh_D10G0354 | 0 | 0 | 1 |
| Gh_D05G3010 | 0 | 0 | 1 |
| Gh_A11G0017 | 0 | 0 | 1 |
| Gh_A03G1711 | 0 | 0 | 1 |
| Gh_A12G0738 | 0 | 0 | 1 |
| Gh_D07G1296 | 0 | 0 | 1 |
| Gh_A03G0694 | 0 | 0 | 1 |
| Gh_A03G0762 | 0 | 0 | 1 |
| Gh_D03G1578 | 0 | 0 | 1 |
| Gh_A06G0765 | 0 | 0 | 1 |
| Gh_D06G2329 | 0 | 0 | 1 |
| Gh_D05G2883 | 0 | 0 | 1 |
| Gh_D08G2705 | 0 | 0 | 1 |
| Gh_A05G2242 | 0 | 0 | 1 |
| Gh_D01G1097 | 0 | 0 | 1 |
| Gh_D12G0762 | 0 | 0 | 1 |
| Gh_Sca005822G01 | 0 | 0 | 1 |

**Table S4. Gene fragment for VIGS.** A 350-bp sequence of Gh_D10G1401 is flanked by sequences specific to virus-induced gene silencing vector pTRV2. These flanking sequences target the EcoRI-KpnI cloning site for Gibson assembly, but abolish both sites and create flanking BamHI sites.

| TGGACTTAGATTCTGTGAGTAAGGTTACCGGATCCATGCAACAAACAAATTAACAAAATGAGAAGTGAAGCATTAAGCAAACCTATGGGGCTGACACATGACATACTTCCCAGAGTTTTCCCTCATTATTACTCCTGGATCAACTTATACGGAAAGAACTATCTTTCTTGGGACGGTGTTCAAGCTCAGGTGGTGACTACTGACCCAGAACTAATAAAAGAGGTTCTTAAAAATAGTGAACAAACTTTTCGGAAAAGGGAACCCCCGATCTATGTTGGCAGGCTATTGGGGGATGGGCTTGTTACAACTGAAGGAGAAAAATGGGCGAAGAAGAGGAAGTTGGCCAACTATGCTTTCCATGGGGAGAGTTTAAAGAACATGACTCGGATCCGTACCGAGCTCACGCGTCTCGAGGCCC |
| --- |

**Table S5. RT-qPCR Primers used in this study.**

| A01G1260_F | TGTGTCTATGCTTAAAGAGGCTGT |
| --- | --- |
| A02G0755_F | ATTCTTTTGGGTTCACAACACTGG |
| A03G0667_F | ACTCCCTAGAGAATCCATGGAAGA |
| A04G0461_F | ATTTGGCTTTGTAACAATGGGGAG |
| A04G0807_F | ACCTCCTCCTTTCTCCTGATAGAA |
| A05G1986_F | AGAGGTAATCTACAGAGGCTCCTT |
| A05G2246_F | CCATTGTTAGTACCTCACGAGTCA |
| A06G0039_F | CAGAGTCTTCTCACACCAGTAACA |
| A11G1753_F | TTGCAGCCATTGATGGATTTCTTT |
| A11G3200_F | CTTTAACAACTTCCCCAAAGGACC |
| A12G0783_F | TCACCATGAAGAAATCAATGCCAC |
| D02G0799_F | ATTCTTTTGGGTTCACAACACTGG |
| D02G1732_F | GGTACCATTGTCCACTGGAGTTAT |
| D03G0963_F | CAATTGACTTCAAGGGGCAAGATT |
| D05G0703_F | ACTTAACAATCAACATGGAGGGAA |
| D05G2142_F | AACCTTGGATTCTGGGATTTCTGA |
| D06G2116_F | TTGCAGAGATGCTGGAAAAAGAAG |
| D06G2275_F | TGGATCGGATGGTTGAAATGATCT |
| D10G1801_F | TCAAAAACCAACCCAAGTTTCCAA |
| D11G1912_F | TTGCAGCCATTGATGGATTTCTTT |
| A01G1260_R | GTAGGTAACTCCAGTTTGTCCGAT |
| A02G0755_R | TGTAAGGCTTTAAACAACCAGCAG |
| A03G0667_R | ATTCTGGATCTCTTCCTATTGCCC |
| A04G0461_R | TTACTTCCCTCTCACCTCCTTTTG |
| A04G0807_R | AAGGAAGACAGCAAAGAGTAGGAG |
| A05G1986_R | TATCGCTCCCTTCAAGTTCTTCTC |
| A05G2246_R | CCCAATTGTTGGGATCGTTTTGTA |
| A06G0039_R | TGCTGTTGATAATGATGGTATTGACC |
| A11G1753_R | GCAACCGAAATTTTCTTGGGAAAC |
| A11G3200_R | TGTTTTCTCCACAAATCCATGTCG |
| A12G0783_R | ACCTCTCTTATTCCCAAACTGTCC |
| D02G0799_R | TGTAAGGCTTTAAACAACCAGCAG |
| D02G1732_R | TATCAGGGTCTACTGCTTCCACTA |
| D03G0963_R | CTCCCAATCAAAGCTATGAAGCAG |
| D05G0703_R | GAACAGACGATGGTGAGTTTACTT |
| D05G2142_R | TCAGCTTCCTGTTTGAGTTTTAAT |
| D06G2116_R | CTTCTCAATCAAACCAAGGACACC |
| D06G2275_R | CTCTGCCCTTTGTTACTGTGTCTA |
| D10G1801_R | TAATTGGACAATGGAAGCAACCAC |
| D11G1912_R | GCAACCGAAATTTTCTTGGGAAAC |

**Table S6. Multiple alignment of Gh_D10G1401 from Paymaster HS26 and wild-type cotton, including potential homeologous region on Chr A1.** Full length coding sequences for Gh_D10G1401 are aligned to the genomic region from Chr D10 and homeologous region from Chr A10. Primers that were used to score the 1-bp insertion in HS26 and TFS sensitive plants are highlighted and labeled as in Table S1. The location of a frameshift deletion in the A10 homeologous sequence is highlighted and labeled A10:54,681,293.

| HS26-Gh_D10G1401 .......... .......... .......... .......... ..........  WT-Gh_D10G1401 .......... .......... .......... .......... ..........  D10:28455640-28458081 TTAACAAAAA GAAAATGAGA ATTTGTGTAT AGAAATAGTA TAAGGGCAAA  A10:54679583-CDS .......... .......... .......... .......... ..........  A10:54679583-54682086 -RC TTAATAAAAA GAAAATGAGA ATTTGTGTAT AGAAATAGTA TAAGGGCAAA  HS26-Gh_D10G1401 .......... .......... .......... .......... ..........  WT-Gh_D10G1401 .......... .......... .......... .......... ..........  D10:28455640-28458081 TAAGTACTAA ATTTTTTACT ATGATTTCTC AATTGTTTTC CTTATCCACA  A10:54679583-CDS .......... .......... .......... .......... ..........  A10:54679583-54682086 -RC TAAGTACTAA TTTTTTT.CT GTGATTTCTC AATTGTTTTC CTTATCCACA  HS26-Gh_D10G1401 .......... .......... .......... .......... ..........  WT-Gh_D10G1401 .......... .......... .......... .......... ..........  D10:28455640-28458081 AAAATACAGG CAAGATATGT CAGTGATCCA TCGTGAAAAC AACTTAACCC  A10:54679583-CDS .......... .......... .......... .......... ..........  A10:54679583-54682086 -RC AAAATACAGG CAAGATACGT GAGTGGTCCA TCGTGAAAAC AACTTAACCC  HS26-Gh_D10G1401 .......... .......... .......... .......... ..........  WT-Gh_D10G1401 .......... .......... .......... .......... ..........  D10:28455640-28458081 AATCAATCCT CCATTTATAG GAGCCTCGAT CTGAATTCAT TTTCAAGATC  A10:54679583-CDS .......... .......... .......... .......... ..........  A10:54679583-54682086 -RC AATCAATCCT CCATTTATAG GAGCCTCGAT CTGAATTCAT TTTCAAGATC  HS26-Gh_D10G1401 ........AT GATGGATGTG AGGGTGAAGC TTTTAATCCT TTTGGCAACC  WT-Gh_D10G1401 ........AT GATGGATGTG AGGGTGAAGC TTTTAATCCT TTTGGCAACC  D10:28455640-28458081 AAAAGGAAAT GATGGATGTG AGGGTGAAGC TTTTAATCCT TTTGGCAACC  A10:54679583-CDS .......... .ATGGATGTG AGGGTGAAGC TTTTAATCCT TTTGGCAACC  A10:54679583-54682086 -RC AAAAGGAAAC GATGGATGTG AGGGTGAAGC TTTTAATCCT TTTGGCAACC  HS26-Gh_D10G1401 TCACTCTTGA TTTACTCGCT CATAGTTTTA CTCAAAGTCC TGTATGACTA  WT-Gh_D10G1401 TCACTCTTGA TTTACTCGCT CATAGTTTTA CTCAAAGTCC TGTATGACTA  D10:28455640-28458081 TCACTCTTGA TTTACTCGCT CATAGTTTTA CTCAAAGTCC TGTATGACTA  A10:54679583-CDS TCGCTCTTGA TTTACTCGCT CATAGTTTTA CTCAAAGTCC TGTATGACTA  A10:54679583-54682086 -RC TCGCTCTTGA TTTACTCGCT CATAGTTTTA CTCAAAGTCC TGTATGACTA  D10_2845_F  HS26-Gh_D10G1401 CTGGTGGGTG CCTCTCCGTA TACAACACTT CTTGAATTCA CAAGGACTTA  WT-Gh_D10G1401 CTGGTGGGTG CCTCTCCGTA TACAACACTT CTTGAATTCA CAAGGACTTA  D10:28455640-28458081 CTGGTGGGTG CCTCTCCGTA TACAACACTT CTTGAATTCA CAAGGACTTA  A10:54679583-CDS CTGGTGGGTG CCTCTCCGTA TACAACACTT CTTGAATTCA CAAGGACTTA  A10:54679583-54682086 -RC CTGGTGGGTG CCTCTCCGTA TACAACACTT CTTGAATTCA CAAGGACTTA  D10_2845_R2  HS26-Gh_D10G1401 AAaGGGCCTC CTTACAAGTT CATCCATGGA TGCAACAAAC AAATTAACAA  WT-Gh_D10G1401 AA.GGGCCTC CTTACAAGTT CATCCATGGA TGCAACAAAC AAATTAACAA  D10:28455640-28458081 AA.GGGCCTC CTTACAAGTT CATCCATGGA TGCAACAAAC AAATTAACAA  A10:54679583-CDS GA.GGGCCTC CTTACAAGTT CATCCATGGA TGCAACAAAC AAATCAACAA  A10:54679583-54682086 -RC GA.GGGCCTC CTTACAAGTT CATCCATGGA TGCAACAAAC AAATCAACAA  HS26-Gh_D10G1401 AATGAGAAGT GAAGCATTAA GCAAACCTAT GGGGCTGACA CATGACATAC  WT-Gh_D10G1401 AATGAGAAGT GAAGCATTAA GCAAACCTAT GGGGCTGACA CATGACATAC  D10:28455640-28458081 AATGAGAAGT GAAGCATTAA GCAAACCTAT GGGGCTGACA CATGACATAC  A10:54679583-CDS AATGAGAAGT GAAGCATTAA GCAAACCTAT GGGGCTGACA CATGACATAC  A10:54679583-54682086 -RC AATGAGAAGT GAAGCATTAA GCAAACCTAT GGGGCTGACA CATGACATAC  HS26-Gh_D10G1401 TTCCCAGAGT TTTCCCTCAT TATTACTCCT GGATCAACTT ATACGG....  WT-Gh_D10G1401 TTCCCAGAGT TTTCCCTCAT TATTACTCCT GGATCAACTT ATACGG....  D10:28455640-28458081 TTCCCAGAGT TTTCCCTCAT TATTACTCCT GGATCAACTT ATACGGTGAG  A10:54679583-CDS TTCCCAGAGT TTTCCCTCAT TATTACTCCT GGATCAACTT ATACGG....  A10:54679583-54682086 -RC TTCCCAGAGT TTTCCCTCAT TATTACTCCT GGATCAACTT ATACGGTGAG  D10_2845_R1  HS26-Gh_D10G1401 .......... .......... .......... .......... ..........  WT-Gh_D10G1401 .......... .......... .......... .......... ..........  D10:28455640-28458081 TGACAAACAT CAAATCAAAT GTATTTTTTT TCACTATGAG TCTAACTTCT  A10:54679583-CDS .......... .......... .......... .......... ..........  A10:54679583-54682086 -RC TGACAAACAT CAAATCAAAT GC.TTTTTTT TCTCTATGAT TCTAGCTTCT  HS26-Gh_D10G1401 .......... .......... .......... .......... ..........  WT-Gh_D10G1401 .......... .......... .......... .......... ..........  D10:28455640-28458081 AGTTTCCAGA AATAATTG.. .......... .......... ..........  A10:54679583-CDS .......... .......... .......... .......... ..........  A10:54679583-54682086 -RC AGTTTCCAGA AATAATTGCA CCATGTTTGG TTGGGAGTAA TGGCTAATCC  HS26-Gh_D10G1401 .......... .......... .......... .......... ..........  WT-Gh_D10G1401 .......... .......... .......... .......... ..........  D10:28455640-28458081 .......... .......... .......... .......... ..........  A10:54679583-CDS .......... .......... .......... .......... ..........  A10:54679583-54682086 -RC ATTACACCCC GATTCGGTGG CCCCACCCAA TACGTCGTTT GGTTCACCGT  HS26-Gh_D10G1401 .......... .......... .......... .........A AAGAACTATC  WT-Gh_D10G1401 .......... .......... .......... .........A AAGAACTATC  D10:28455640-28458081 ..GTTTTTTC TTTTCTTTTT TTAAATTGAG TTGATTAGGA AAGAACTATC  A10:54679583-CDS .......... .......... .......... .........A AAGAACTATC  A10:54679583-54682086 -RC TGGTTTTTTC TTTTCTTGTT TTCAATTGAG TTGATTAGGA AAGAACTATC  HS26-Gh_D10G1401 TTTCTTGGGA CGGTGTTCAA GCTCAGGTGG TGACTACTGA CCCAGAACTA  WT-Gh_D10G1401 TTTCTTGGGA CGGTGTTCAA GCTCAGGTGG TGACTACTGA CCCAGAACTA  D10:28455640-28458081 TTTCTTGGGA CGGTGTTCAA GCTCAGGTGG TGACTACTGA CCCAGAACTA  A10:54679583-CDS TTTCTTGGGA CGGTGCTCAA GCTCAGGTGG TGACTACTGA CCCAGAACTA  A10:54679583-54682086 -RC TTTCTTGGGA CGGTGCTCAA GCTCAGGTGG TGACTACTGA CCCAGAACTA  A10:54,681,293  HS26-Gh_D10G1401 ATAAAAGAGG TTCTTAAAAA TAGTGAACAA ACTTTTCGGA AAAGGGAACC  WT-Gh_D10G1401 ATAAAAGAGG TTCTTAAAAA TAGTGAACAA ACTTTTCGGA AAAGGGAACC  D10:28455640-28458081 ATAAAAGAGG TTCTTAAAAA TAGTGAACAA ACTTTTCGGA AAAGGGAACC  A10:54679583-CDS ATGAAAGAGG TTCTTAAAAA TAGTGAACAA ACTTTTCCGA AAAGGGA.CC  A10:54679583-54682086-RC ATGAAAGAGG TTCTTAAAAA TAGTGAACAA ACTTTTCCGA AAAGGGA.CC  HS26-Gh_D10G1401 CCCGATCTAT GTTGGCAGGC TATTGGGGGA TGGGCTTGTT ACAACTGAAG  WT-Gh_D10G1401 CCCGATCTAT GTTGGCAGGC TATTGGGGGA TGGGCTTGTT ACAACTGAAG  D10:28455640-28458081 CCCGATCTAT GTTGGCAGGC TATTGGGGGA TGGGCTTGTT ACAACTGAAG  A10:54679583-CDS CCCTATTTAT GTTGGCAGGC TATTGGGGAA TGGGCTTGTT ACAACTGAAG  A10:54679583-54682086 -RC CCCTATTTAT GTTGGCAGGC TATTGGGGAA TGGGCTTGTT ACAACTGAAG  HS26-Gh_D10G1401 GAGAAAAATG GGCGAAGAAG AGGAAGTTGG CCAACTATGC TTTCCATGGG  WT-Gh_D10G1401 GAGAAAAATG GGCGAAGAAG AGGAAGTTGG CCAACTATGC TTTCCATGGG  D10:28455640-28458081 GAGAAAAATG GGCGAAGAAG AGGAAGTTGG CCAACTATGC TTTCCATGGG  A10:54679583-CDS GAGAAAAATG GGCGAAGAAG AGGAAGTTGG CCAACTATGT TTTCCATGGG  A10:54679583-54682086 -RC GAGAAAAATG GGCGAAGAAG AGGAAGTTGG CCAACTATGT TTTCCATGGG  HS26-Gh_D10G1401 GAGAGTTTAA AG........ .......... .......... ..........  WT-Gh_D10G1401 GAGAGTTTAA AG........ .......... .......... ..........  D10:28455640-28458081 GAGAGTTTAA AGGTAAAATA TTGTGGGTGA TTTGAGTTAT TAATTCTTGG  A10:54679583-CDS GAGAGTTTAA AG........ .......... .......... ..........  A10:54679583-54682086 -RC GAGAGTTTAA AGGTAAAATC TTGTGGGTGA TTTGAGTTAT TAATTCTTGG  HS26-Gh_D10G1401 .......... .......... .......... ....AACATG ACTCCAGCAG  WT-Gh_D10G1401 .......... .......... .......... ....AACATG ACTCCAGCAG  D10:28455640-28458081 TTACATTTAA CACATTGGAG GATGGTTTCG GCAGAACATG ACTCCAGCAG  A10:54679583-CDS .......... .......... .......... ....AACATG ACTCCAGCAG  A10:54679583-54682086 -RC TTACATTTAA CACATTGGAG GATGGTTTCG GCAGAACATG ACTCCAGCAG  HS26-Gh_D10G1401 TAATTGCCAG CGTTGAAACA ATGCTAGACA AGTGGAAAGA CAAAGAAGGA  WT-Gh_D10G1401 TAATTGCCAG CGTTGAAACA ATGCTAGACA AGTGGAAAGA CAAAGAAGGA  D10:28455640-28458081 TAATTGCCAG CGTTGAAACA ATGCTAGACA AGTGGAAAGA CAAAGAAGGA  A10:54679583-CDS TAATTGCCAG CGTTGAAACA ATGCTAGACA AGTGGCAAGA GAAAGAAGGA  A10:54679583-54682086 -RC TAATTGCCAG CGTTGAAACA ATGCTAGACA AGTGGCAAGA GAAAGAAGGA  HS26-Gh_D10G1401 AAAGAGATCG AAGCATTCCA AGAATTTAGA ATCTTGACTT CAGAAGTCAT  WT-Gh_D10G1401 AAAGAGATCG AAGCATTCCA AGAATTTAGA ATCTTGACTT CAGAAGTCAT  D10:28455640-28458081 AAAGAGATCG AAGCATTCCA AGAATTTAGA ATCTTGACTT CAGAAGTCAT  A10:54679583-CDS GAAGAGATCG AAGCGTTCCA AGGATTTAGA TTGTTGACTT CAGAAGTCAT  A10:54679583-54682086 -RC GAAGAGATCG AAGCGTTCCA AGGATTTAGA TTGTTGACTT CAGAAGTCAT  HS26-Gh_D10G1401 ATCAAGAACA GCTTTTGGTA GCAGTTACTT CGAAGGAGAG AAGATTTTTT  WT-Gh_D10G1401 ATCAAGAACA GCTTTTGGTA GCAGTTACTT CGAAGGAGAG AAGATTTTTT  D10:28455640-28458081 ATCAAGAACA GCTTTTGGTA GCAGTTACTT CGAAGGAGAG AAGATTTTTT  A10:54679583-CDS ATCAAGAACA GCTTTTGGTA GCAGTTACTT GGAAGGAGAG AAGATCTTTT  A10:54679583-54682086 -RC ATCAAGAACA GCTTTTGGTA GCAGTTACTT GGAAGGAGAG AAGATCTTTT  HS26-Gh_D10G1401 ACATGTTGCA GAAGCTCGCA GATATTGTGA GCCGCAATTC AAACAAGTCT  WT-Gh_D10G1401 ACATGTTGCA GAAGCTCGCA GATATTGTGA GCCGCAATTC AAACAAGTCT  D10:28455640-28458081 ACATGTTGCA GAAGCTCGCA GATATTGTGA GCCGCAATTC AAACAAGTCT  A10:54679583-CDS ACATGTTGCA GAAGCTCGCA GATATTGTGA GCCGCAATAC AAACAAGTCT  A10:54679583-54682086 -RC ACATGTTGCA GAAGCTCGCA GATATTGTGA GCCGCAATAC AAACAAGTCT  HS26-Gh_D10G1401 AGGATTCCAA TCCTCAG... .......... .......... ..........  WT-Gh_D10G1401 AGGATTCCAA TCCTCAG... .......... .......... ..........  D10:28455640-28458081 AGGATTCCAA TCCTCAGGTA TGTTTGTTTA TGTTGGTTGA GTGTTAAGGA  A10:54679583-CDS AGGATTCCAA TCCTCAG... .......... .......... ..........  A10:54679583-54682086 -RC AGGATTCCAA TCCTCAGGTA TGTTTGTTTA TGTTGGTTGG GTGTTAAGGA  HS26-Gh_D10G1401 .......... .......... .......... .......... ..........  WT-Gh_D10G1401 .......... .......... .......... .......... ..........  D10:28455640-28458081 GATTAGTACA GGTCTTGAGT TTGAACCTTG TTACTGTTTG ATGACATCGA  A10:54679583-CDS .......... .......... .......... .......... ..........  A10:54679583-54682086 -RC GATTAGTACA GGTCTTGAAT TTGAACCTTG TTACTGTTTG ATGGCATCGA  HS26-Gh_D10G1401 .......... ...CAAGTTT TGGAAAACTG ACGATGACAT TGAGTCAGAG  WT-Gh_D10G1401 .......... ...CAAGTTT TGGAAAACTG ACGATGACAT TGAGTCAGAG  D10:28455640-28458081 TTTGATTTTG CAGCAAGTTT TGGAAAACTG ACGATGACAT TGAGTCAGAG  A10:54679583-CDS .......... ...CAAGTTT TGGAAAACTG ACGATGACAT AGAGTCAGAA  A10:54679583-54682086 -RC TTTGATTTTG CAGCAAGTTT TGGAAAACTG ACGATGACAT AGAGTCAGAA  HS26-Gh_D10G1401 AAACTTGCTA GAGAAATACA AGATTTGGTT ATAGAGATTG TGAAGAAAAG  WT-Gh_D10G1401 AAACTTGCTA GAGAAATACA AGATTTGGTT ATAGAGATTG TGAAGAAAAG  D10:28455640-28458081 AAACTTGCTA GAGAAATACA AGATTTGGTT ATAGAGATTG TGAAGAAAAG  A10:54679583-CDS AAACTTGCTA GAGAAATACA AGATTTGGTG ATAGAGATTG TGAAGAAAAG  A10:54679583-54682086 -RC AAACTTGCTA GAGAAATACA AGATTTGGTG ATAGAGATTG TGAAGAAAAG  HS26-Gh_D10G1401 AGAAAACAAA GTTTCGAGTG GAGGAGCAGA GAGCTTCGGC AGTGATTTTC  WT-Gh_D10G1401 AGAAAACAAA GTTTCGAGTG GAGGAGCAGA GAGCTTCGGC AGTGATTTTC  D10:28455640-28458081 AGAAAACAAA GTTTCGAGTG GAGGAGCAGA GAGCTTCGGC AGTGATTTTC  A10:54679583-CDS AGAAAACAAA GTTGCGAGTG GAGGAGCAGA GAGCTTCGGC TGTGATTTTC  A10:54679583-54682086 -RC AGAAAACAAA GTTGCGAGTG GAGGAGCAGA GAGCTTCGGC TGTGATTTTC  HS26-Gh_D10G1401 TGGGATTATT AGTAAAAGCG TTTAATAATT CAGATGAAAA AAACAAGATT  WT-Gh_D10G1401 TGGGATTATT AGTAAAAGCG TTTAATAATT CAGATGAAAA AAACAAGATT  D10:28455640-28458081 TGGGATTATT AGTAAAAGCG TTTAATAATT CAGATGAAAA AAACAAGATT  A10:54679583-CDS TGGGATTATT AGTAAAAGCG TTTAATAATC CAGATGAAAA AAACAAGATT  A10:54679583-54682086 -RC TGGGATTATT AGTAAAAGCG TTTAATAATC CAGATGAAAA AAACAAGATT  HS26-Gh_D10G1401 TCAATGGAAG ATTTGGTTGA TGAGTGCAAA ACATTTTATT TTGCCGGACA  WT-Gh_D10G1401 TCAATGGAAG ATTTGGTTGA TGAGTGCAAA ACATTTTATT TTGCCGGACA  D10:28455640-28458081 TCAATGGAAG ATTTGGTTGA TGAGTGCAAA ACATTTTATT TTGCCGGACA  A10:54679583-CDS TCAATGGAAG ATTTGGTTGA TGAGTGCAAA ACATTTTATT TTGCCGGACA  A10:54679583-54682086 -RC TCAATGGAAG ATTTGGTTGA TGAGTGCAAA ACATTTTATT TTGCCGGACA  HS26-Gh_D10G1401 AGAAACTGTT AACGCCTCAC TTGCTTGGGC AGTGCTGGTT TTGGCTATCC  WT-Gh_D10G1401 AGAAACTGTT AACGCCTCAC TTGCTTGGGC AGTGCTGGTT TTGGCTATCC  D10:28455640-28458081 AGAAACTGTT AACGCCTCAC TTGCTTGGGC AGTGCTGGTT TTGGCTATCC  A10:54679583-CDS AGAAACTGTT AATGCCTCGC TTGCTTGGGC AGTGCTGGTT TTAGCTATCC  A10:54679583-54682086 -RC AGAAACTGTT AATGCCTCGC TTGCTTGGGC AGTGCTGGTT TTAGCTATCC  HS26-Gh_D10G1401 ATAGAGATTG GCAAGACAAA GCAAGAAGAG AGGTGATGGA GATATTCGGT  WT-Gh_D10G1401 ATAGAGATTG GCAAGACAAA GCAAGAAGAG AGGTGATGGA GATATTCGGT  D10:28455640-28458081 ATAGAGATTG GCAAGACAAA GCAAGAAGAG AGGTGATGGA GATATTCGGT  A10:54679583-CDS ATAGAGATTG GCAAGACAAA GCAAGAAGAG AGGTGATGGA GATATTCGGT  A10:54679583-54682086 -RC ATAGAGATTG GCAAGACAAA GCAAGAAGAG AGGTGATGGA GATATTCGGT  HS26-Gh_D10G1401 AAACAAAATC CACACTCCGA AGGCCTTGCC GAACTCAAAA TC........  WT-Gh_D10G1401 AAACAAAATC CACACTCCGA AGGCCTTGCC GAACTCAAAA TC........  D10:28455640-28458081 AAACAAAATC CACACTCCGA AGGCCTTGCC GAACTCAAAA TCGTAAGCAA  A10:54679583-CDS AGCCAATATC CACACTTCGA AGGCCTTGCC AAACTCAAAA TC........  A10:54679583-54682086 -RC AGCCAATATC CACACTTCGA AGGCCTTGCC AAACTCAAAA TCGTAAGCAA  HS26-Gh_D10G1401 .......... .......... .......... .......... ..........  WT-Gh_D10G1401 .......... .......... .......... .......... ..........  D10:28455640-28458081 ATATGAT..A GCTTACTTGT ATTATTGTTT TCCGTTTAAA TGAAACTGAA  A10:54679583-CDS .......... .......... .......... .......... ..........  A10:54679583-54682086 -RC ATTGGCTTAA CTGTACCTGT ATTGTTGTTT CTCATTTAAA TGAAACTGAA  HS26-Gh_D10G1401 .......... .......... .......... .......ATG AGCATGATCA  WT-Gh_D10G1401 .......... .......... .......... .......ATG AGCATGATCA  D10:28455640-28458081 ATCTAACTTA TGTTATCCAT CCCCTTTTTG CAAACAGATG AGCATGATCA  A10:54679583-CDS .......... .......... .......... .......ATG AGCATGATCA  A10:54679583-54682086 -RC ATATAACTT. .......... .......TTG CAAACAGATG AGCATGATCA  HS26-Gh_D10G1401 TTAATGAAAC TCTACGATTG TATACTCCCT TAAATGGGAT GGTAAGAAGA  WT-Gh_D10G1401 TTAATGAAAC TCTACGATTG TATACTCCCT TAAATGGGAT GGTAAGAAGA  D10:28455640-28458081 TTAATGAAAC TCTACGATTG TATACTCCCT TAAATGGGAT GGTAAGAAGA  A10:54679583-CDS TTAATGAAAC TCTACGATTG TATACTCCCT TAAACGGGTT GTTAAGAAGA  A10:54679583-54682086 -RC TTAATGAAAC TCTACGATTG TATACTCCCT TAAACGGGTT GTTAAGAAGA  HS26-Gh_D10G1401 GCTAGAAGAC AAGTTCAAGT GGGAAAGCTA GTCTTGCCTG CTGAAGTAGA  WT-Gh_D10G1401 GCTAGAAGAC AAGTTCAAGT GGGAAAGCTA GTCTTGCCTG CTGAAGTAGA  D10:28455640-28458081 GCTAGAAGAC AAGTTCAAGT GGGAAAGCTA GTCTTGCCTG CTGAAGTAGA  A10:54679583-CDS GCTACAAGAC AAGTTCAAGT GGGAAAGCTA GTTTTGCCTG CTAAAGTAGA  A10:54679583-54682086 -RC GCTACAAGAC AAGTTCAAGT GGGAAAGCTA GTTTTGCCTG CTAAAGTAGA  HS26-Gh_D10G1401 TGTTTTGATC CCAAACATGG CACTTCACCA TGACCCTGAG TTATGGGGAG  WT-Gh_D10G1401 TGTTTTGATC CCAAACATGG CACTTCACCA TGACCCTGAG TTATGGGGAG  D10:28455640-28458081 TGTTTTGATC CCAAACATGG CACTTCACCA TGACCCTGAG TTATGGGGAG  A10:54679583-CDS AGTTTTGATC CCAAACATGG CACTTCACCA TGACCCTCAG TTATGGGGAG  A10:54679583-54682086 -RC AGTTTTGATC CCAAACATGG CACTTCACCA TGACCCTCAG TTATGGGGAG  HS26-Gh_D10G1401 ACGATGCCCA TGTTTTCAAA CCGGAGAGGT TCGAGGAAGG GATCGCGAAA  WT-Gh_D10G1401 ACGATGCCCA TGTTTTCAAA CCGGAGAGGT TCGAGGAAGG GATCGCGAAA  D10:28455640-28458081 ACGATGCCCA TGTTTTCAAA CCGGAGAGGT TCGAGGAAGG GATCGCGAAA  A10:54679583-CDS ACGATGCCCA TGTTTTCAAA CCGGAGAGGT TCGAGGAAGG GATCGCAAAA  A10:54679583-54682086 -RC ACGATGCCCA TGTTTTCAAA CCGGAGAGGT TCGAGGAAGG GATCGCAAAA  HS26-Gh_D10G1401 GCTACCAAGT ACAATGCGGC TGCATTTATT CCTTTCGGAA TGGGACCTCG  WT-Gh_D10G1401 GCTACCAAGT ACAATGCGGC TGCATTTATT CCTTTCGGAA TGGGACCTCG  D10:28455640-28458081 GCTACCAAGT ACAATGCGGC TGCATTTATT CCTTTCGGAA TGGGACCTCG  A10:54679583-CDS GCTACCAGGT ACAATGCTGC TGCATTTATT CCTTTCGGAA TGGGACCTCG  A10:54679583-54682086 -RC GCTACCAGGT ACAATGCTGC TGCATTTATT CCTTTCGGAA TGGGACCTCG  HS26-Gh_D10G1401 AGCTTGTGTC GGGATGACCT TTGCAATCAA TGAAACAAAG ACTGCTCTTT  WT-Gh_D10G1401 AGCTTGTGTC GGGATGACCT TTGCAATCAA TGAAACAAAG ACTGCTCTTT  D10:28455640-28458081 AGCTTGTGTC GGGATGACCT TTGCAATCAA TGAAACAAAG ACTGCTCTTT  A10:54679583-CDS AGCTTGTGTC GGGATGACCT TTGCAATCAA TGAAACAAAG ACTGCTCTTT  A10:54679583-54682086 -RC AGCTTGTGTC GGGATGACCT TTGCAATCAA TGAAACAAAG ACTGCTCTTT  HS26-Gh_D10G1401 CCATGATTCT ACAAAGATAC ACCATTTCTC TCTCCCCTGC GTATGTTCAC  WT-Gh_D10G1401 CCATGATTCT ACAAAGATAC ACCATTTCTC TCTCCCCTGC GTATGTTCAC  D10:28455640-28458081 CCATGATTCT ACAAAGATAC ACCATTTCTC TCTCCCCTGC GTATGTTCAC  A10:54679583-CDS CCATGATTCT ACAAAGATAC ACCATTTCTC TCTCCCCTGC CTATGTTCAC  A10:54679583-54682086 -RC CCATGATTCT ACAAAGATAC ACCATTTCTC TCTCCCCTGC CTATGTTCAC  HS26-Gh_D10G1401 TCACCCGCAC CTCGTATCAC AGTTCAACCA CAACATGGGA TTCAAGTTAT  WT-Gh_D10G1401 TCACCCGCAC CTCGTATCAC AGTTCAACCA CAACATGGGA TTCAAGTTAT  D10:28455640-28458081 TCACCCGCAC CTCGTATCAC AGTTCAACCA CAACATGGGA TTCAAGTTAT  A10:54679583-CDS TCACCCACAC CTCGTATCAC AGTTCAACCA CAACATGGGA TTCAAGTTGT  A10:54679583-54682086 -RC TCACCCACAC CTCGTATCAC AGTTCAACCA CAACATGGGA TTCAAGTTGT  HS26-Gh_D10G1401 GCTCAATTCA CTGTCTAATG ATGCTTAA.. .......... ..........  WT-Gh_D10G1401 GCTCAATTCA CTGTCTAATG ATGCTTAA.. .......... ..........  D10:28455640-28458081 GCTCAATTCA CTGTCTAATG ATGCTTAAAC TAGGCGCTGT TGATCTGTAA  A10:54679583-CDS ACTGAACTCA CTGTCTAATG ATGCTTAA.. .......... ..........  A10:54679583-54682086 -RC ACTGAACTCA CTGTCTAATG ATGCTTAAAC TAGGCGCTGT TGATCTGTAA  HS26-Gh_D10G1401 .......... .......... .......... .......... ..........  WT-Gh_D10G1401 .......... .......... .......... .......... ..........  D10:28455640-28458081 CGCCGTAATC AATGAGTAAA CTCTGGTGTC GGACTCTAGT AATGTTTGTT  A10:54679583-CDS .......... .......... .......... .......... ..........  A10:54679583-54682086 -RC CGCCGTAATC AATAAGTAAA CTCTAGTGTC GGACTCTAGT AATGTTTGTT  HS26-Gh_D10G1401 .......... .......... .......... .......... ..........  WT-Gh_D10G1401 .......... .......... .......... .......... ..........  D10:28455640-28458081 CAGTTTGACC TGCCATGCAC TTTGCCAATT CCCCATGTTC GCATGTCTGT  A10:54679583-CDS .......... .......... .......... .......... ..........  A10:54679583-54682086 -RC CAGTTTGACC TGCCATGCAC TTTGCCAATT CCCTATGTTC GCATGT...T  HS26-Gh_D10G1401 .......... .......... .......... .......... ..........  WT-Gh_D10G1401 .......... .......... .......... .......... ..........  D10:28455640-28458081 GAATGTTAGG TTGTAGAATT ATTAGAGTTC TTTATTTTCT CTAAAATATA  A10:54679583-CDS .......... .......... .......... .......... ..........  A10:54679583-54682086 -RC GAATGTTAGG TTGTAGAATT ATTCGAGTTC TTTATTTTCT CTAAAATATA  HS26-Gh_D10G1401 .......... .......... .......... .......... ..........  WT-Gh_D10G1401 .......... .......... .......... .......... ..........  D10:28455640-28458081 TGTAAATGCT TCTATTAGAT CCGGAGATCT GATATGAGAA CATATCCAAA  A10:54679583-CDS .......... .......... .......... .......... ..........  A10:54679583-54682086 -RC TGTAAATGCT TCTATTAGAT CCGGAGATCT GATATGAGAA CATATCCAAA  HS26-Gh_D10G1401 .......... .......... .......... .......... ..........  WT-Gh_D10G1401 .......... .......... .......... .......... ..........  D10:28455640-28458081 TATATTAGGA GACTTCAAGA AGTTTAGTTT AATCGTATTG ATTCAATTTG  A10:54679583-CDS .......... .......... .......... .......... ..........  A10:54679583-54682086 -RC TATATTAGGA GACTTCAAGA AGTTTAGTTT AATCGTATTA ATTCAATTTG  HS26-Gh_D10G1401 .......... .......... .......... ..  WT-Gh_D10G1401 .......... .......... .......... ..  D10:28455640-28458081 GTAACATTAC AAGAAAGCAG TAGCATACA. ..  A10:54679583-CDS .......... .......... .......... ..  A10:54679583-54682086 -RC GTAACATTAC AAGAAAGCAG TAGCATACA. .. |
| --- |
