# Supplementary figures and images for: The P450 gene CYP749A16 is required for tolerance to the sulfonylurea herbicide trifloxysulfuron sodium in cotton (Gossypium hirsutum L.)
Source: BMC Plant Biol. 2018 Sep 10;18:186. doi: 10.1186/s12870-018-1414-2 (PMC6131939; doi:10.1186/s12870-018-1414-2)

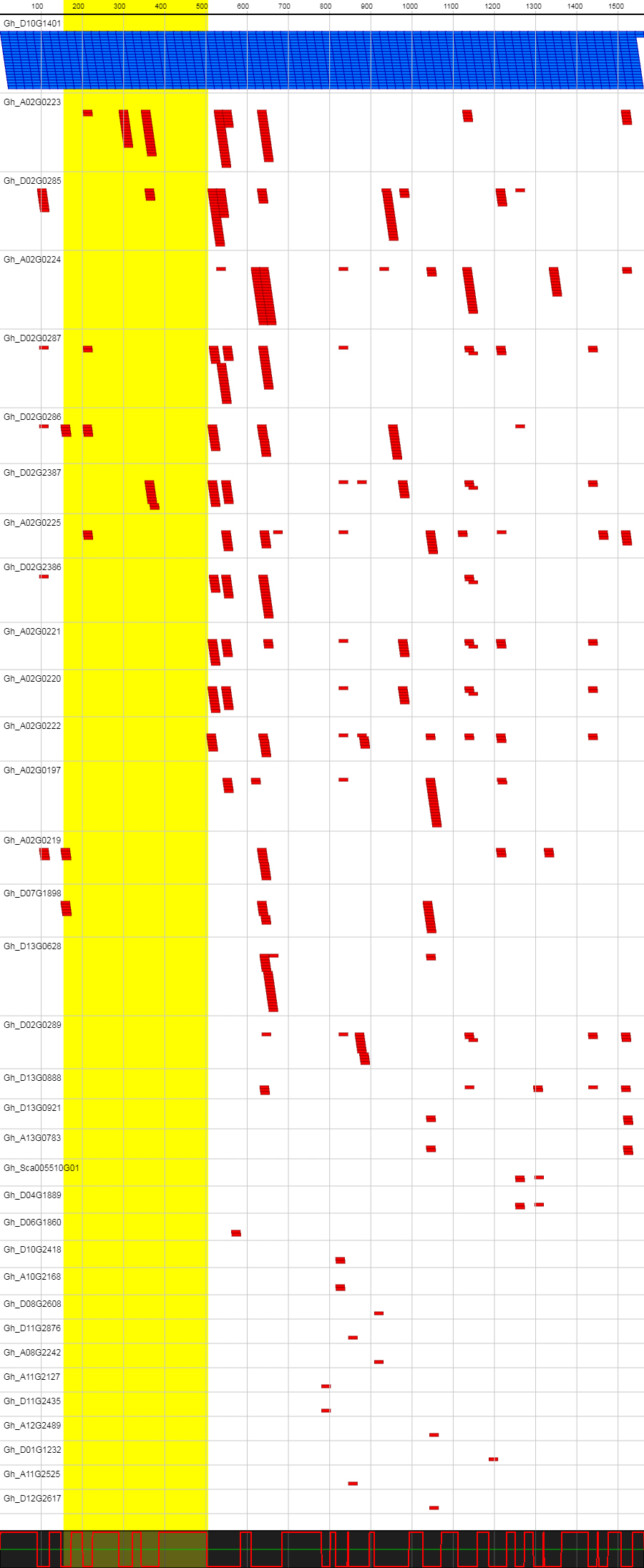

Supplement: Supplementary file 2 — Figure S1. Design of VIGS target sequence. The SGN VIGS Tool software selected the 350-bp targeting sequence (yellow region) from the NBI reference genome for cotton, Gossypium hirsutum cv. TM-1. The full length coding sequence of Gh_D10G1401 was chopped into all 1542 possible 21-mers. These 21-mers were aligned to annotated coding sequences, allowing one mismatch. Off target alignments of 21-mers are shown in red. (JPG 683 kb) [file 12870_2018_1414_MOESM2_ESM.jpg]

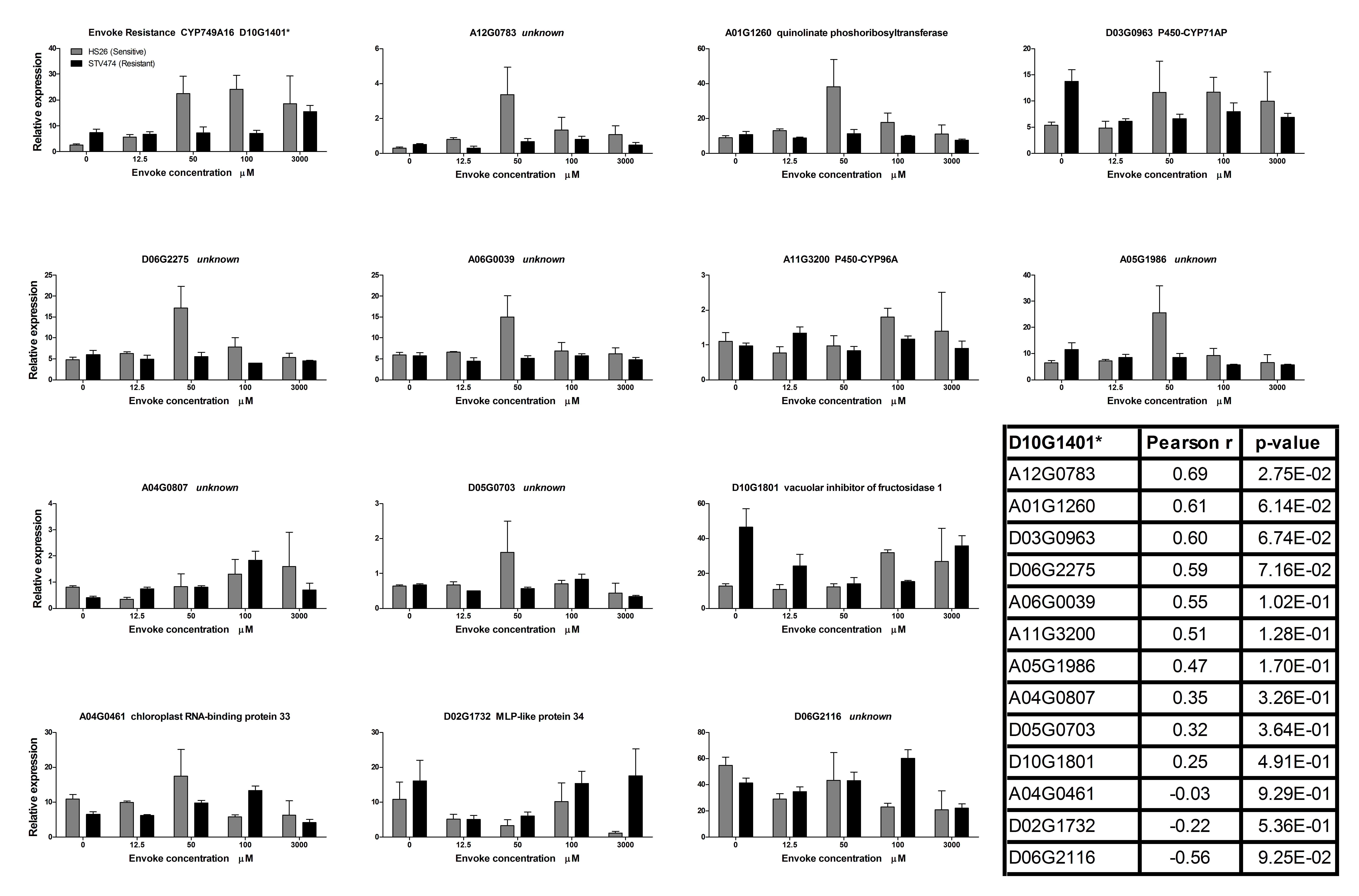

Supplement: Supplementary file 3 — Figure S2. Response of additional genes to TFS application in tolerant and sensitive cotton cultivars. Two-week old cotton plants were exposed to 0, 12.5, 50, 100 or 3000 μM TFS. The tolerant cultivar is STV474 and is shown with black bars, while the sensitive HS26 is grey. Error bars represent standard deviation from six biological replicates. The table shows Pearson’s pairwise correlation in expression patterns between tested genes and CYP749A16 (Gh_D10G1401). See also Fig. 2. (JPG 5645 kb) [file 12870_2018_1414_MOESM3_ESM.jpg]
